# switchde: inference of switch-like differential expression along single-cell trajectories

**DOI:** 10.1093/bioinformatics/btw798

**Published:** 2016-12-30

**Authors:** Kieran R Campbell, Christopher Yau

**Affiliations:** 1 Department of Physiology, Anatomy and Genetics; 2 Wellcome Trust Centre for Human Genetics; 3 Department of Statistics, University of Oxford, Oxford, UK

## Abstract

**Motivation:**

Pseudotime analyses of single-cell RNA-seq data have become increasingly common. Typically, a latent trajectory corresponding to a biological process of interest—such as differentiation or cell cycle—is discovered. However, relatively little attention has been paid to modelling the differential expression of genes along such trajectories.

**Results:**

We present switchde, a statistical framework and accompanying R package for identifying switch-like differential expression of genes along pseudotemporal trajectories. Our method includes fast model fitting that provides interpretable parameter estimates corresponding to how quickly a gene is up or down regulated as well as where in the trajectory such regulation occurs. It also reports a *P*-value in favour of rejecting a constant-expression model for switch-like differential expression and optionally models the zero-inflation prevalent in single-cell data.

**Availability and Implementation:**

The R package switchde is available through the Bioconductor project at https://bioconductor.org/packages/switchde.

**Supplementary information:**

Supplementary data are available at *Bioinformatics* online.

## 1 Introduction

Single-cell RNA-sequencing (scRNA-seq) has transformed biology by providing high-throughput quantification of mRNA abundance in individual cells allowing, amongst other things, the identification of novel cell types and gene expression heterogeneity (Trapnell, 2015). Single-cell pseudotime estimation (Ji and Ji, 2016; Reid and Wernisch, 2016; Shin *et al.*, 2015; Trapnell *et al.*, 2014) has also enabled gene expression profiles to be mapped to a unique value known as the *pseudotime*—a surrogate measure of the cellular state in temporally evolving biological process such as differentiation or cell-cycle.

Once a pseudotime has been assigned to each cell it is possible to identify genes that exhibit a strong pseudotemporal dependence through differential expression testing. An approach first introduced in Trapnell *et al.* (2014) was to regress gene expression on pseudotime using cubic B-spline basis functions with a Tobit likelihood. However, the flexible nonparametric nature of such models may lead to overfitting and may also be difficult to interpret. To our knowledge no other differential-expression-along-pseudotime models have been proposed.

As a solution to these issues we present switchde, a statistical model and accompanying R package for identifying switch-like differential expression analysis along single-cell trajectories. We model sigmoidal expression changes along pseudotime that provides interpretable parameter estimates corresponding to gene regulation strength and timing along with hypothesis testing for differential expression. Our model optionally incorporates zero-inflation for datasets that exhibit high numbers of missing measurements.

## 2 Materials and methods

**Fig. 1.**
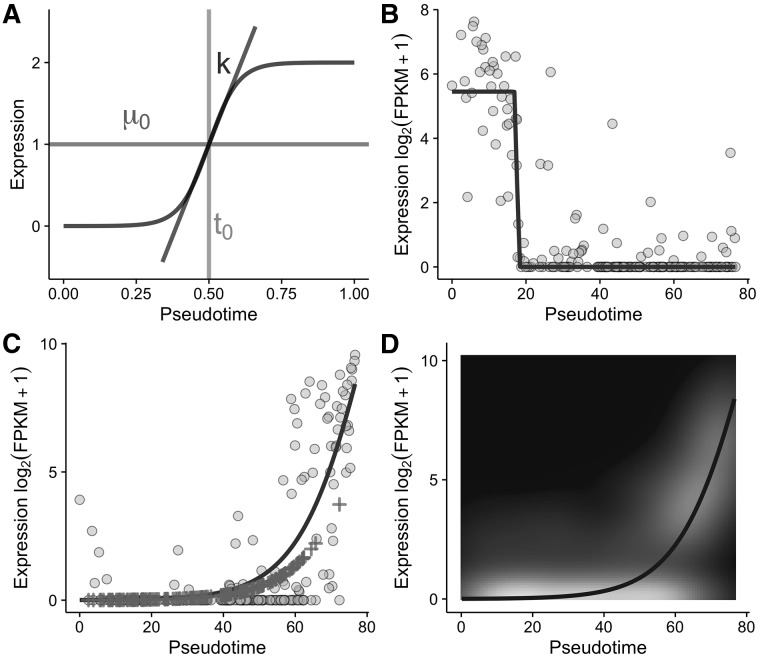
Sigmoidal expression across pseudotime. (**A**) The sigmoid curve as a model of gene expression along single-cell trajectories, parametrized by the average peak expression μ_0_, the activation strength *k* and the activation time *t*_0_. (**B**) An example using the *NDC80* gene from the Trapnell dataset (Trapnell *et al.* (2014)), which had the lowest *P*-value of all genes tested. Gene expression measurements are shown as the grey points with the maximum likelihood sigmoid fit denoted by the dark line. The maximum likelihood parameter estimates were $\mu_{g}^{\left(0\right)}=2.73,\;k_{g}=-8.71$ and $t_{g}^{\left(0\right)}=17.61$. (**C**) Zero-inflated differential expression for the transcription factor *MYOG*. Solid line shows the MLE sigmoidal mean while crosses show imputed gene expression measured as zeroes. (**D**) Posterior predictive density for the zero-inflated model with the solid line denoting MLE sigmoidal mean.

We begin with a *C *×* G* expression matrix Y for *G* genes and *C* cells with column vector $y_{g},g\in 1,\ldots ,G$, that is non-negative and represents gene expression in a form comparable to log ⁡(TPM+1). We define the sigmoid function as $f(t_{c};\mu_{g}^{\left(0\right)},k_{g},t_{g}^{\left(0\right)})=\frac{2\mu_{g}^{\left(0\right)}}{1+\text{exp}\;\left(-k_{g}(t_{c}-t_{g}^{\left(0\right)})\right)}$ where $t_{c},c\in 1,\ldots ,C$ is the latent pseudotime of cell *c*. The parameters (Fig. 1A) may be interpreted as the average peak expression level ($\mu_{g}^{\left(0\right)}$), the *activation strength k_g_* or how quickly a gene is up-or-down regulated and the *activation time* ($t_{g}^{\left(0\right)}$), or where in the trajectory the gene regulation occurs.

We fit the model using gradient-based L-BFGS-B optimization to find maximum likelihood estimates (MLEs) of the parameters (Supplementary Methods). By setting *k_g_* = 0 we identify a nested constant-expression model where $y_{g}∼N(\mu_{g}^{\left(0\right)},\sigma_{g}^{2})$ and so can perform a likelihood ratio test for differential expression, where twice the difference in the log-likelihood MLE between the constant and sigmoidal models asymptotically follows a $\chi^{2}$ distribution with two degrees of freedom.

scRNA-seq data is also known to exhibit a large number of *dropouts* where the expression measurements of low abundance transcripts are zero (Kharchenko *et al.* (2014)). This leads to sparse input matrices for downstream analysis which may violate assumptions of statistical models, such as the Gaussian likelihood above. Therefore, we have also developed an extension for datasets with high dropout rates that incorporates a zero-inflated likelihood similar to Pierson and Yau (2015).

## 3 Results and discussion

We applied switchde to the set of differentiating myoblasts from Trapnell *et al.* (2014). Using the originally published pseudotimes, we removed cells corresponding to contaminating mesenchymal cells and fitted switch-like models for the 11 253 genes expressed in at least 20% of cells with a mean expression of 0.1 FPKM, which took less than a minute on a laptop computer. 2336 genes were found to be significantly differentially expressed at 5% FDR after Benjamini-Hochberg multiple testing correction. The gene with the lowest reported *P*-value was *NDC80* whose expression is plotted in Figure 1B along with the MLE sigmoid fit. The maximum likelihood parameter estimates were $k_{g}=-8.71$, indicating strong down-regulation and $t_{g}^{\left(0\right)}=17.61$, which given the pseudotimes range from 0 to 77 indicates this down-regulation occurs within the first quarter of the trajectory.

We next applied switchde in zero-inflated mode to a subset of genes from the same dataset. While zero-inflated mode accounts for dropout and is thus a less mis-specified model, the Expectation-Maximization algorithm required for inference takes on average an order of magnitude longer. The resulting fit for the transcription factor *MYOG* can be seen in Figure 1C. One advantage of the zero-inflated model is that transcripts that exhibit dropout may be imputed given the pseudotemporal trend, shown by the crosses in the figure. Finally, since switchde specifies a fully generative probabilistic model we can generate a posterior predictive distribution of gene expression over pseudotime. This distribution for *MYOG* is shown in Figure 1D, demonstrating the model is well calibrated with the overall pseudotemporal trend. Further data examples are given in Supplementary Material.

In this paper we have introduced switchde, the first dedicated statistical framework for modelling differential expression over pseudotime. By assuming a parametric model of gene expression along trajectories our model provides interpretable parameter estimates corresponding to gene regulation strength and timing, incorporating zero-inflation that is prevalent in many scRNA-seq datasets. Finally, our model provides hypothesis testing for switch-like differential expression, though in practice this may lead to an inflated false discovery rate due to the assumption that pseudotimes are fixed (Campbell and Yau (2016)).

## Supplementary Material

Supplementary Data
