# Supplementary material for: switchde: inference of switch-like differential expression along single-cell trajectories
Source: Bioinformatics. 2016 Dec 30;33(8):1241–2. doi: 10.1093/bioinformatics/btw798 (PMC5408844; doi:10.1093/bioinformatics/btw798)
Supplement: Supplementary Data [file btw798_supp.pdf]

Supplementary Text, Figures and Methods

Kieran Campbell and Christopher Yau

November 11, 2016

Contents

1 Supplementary text and figures **1**
1.1 Expression profiles of marker genes 1
1.2 Examples of large and small p-values 1
1.3 Tracing activation times along pseudotime 1
1.4 Comparison of zero-inflated and standard models 2

2 Supplementary methods **2**
2.1 Maximum likelihood model fitting 2
2.2 Expectation-Maximisation for zero inflation 4

1 Supplementary text and figures

1.1 Expression profiles of marker genes

In [Trapnell *et al.*, 2014] the genes *CDK1* and *ID1* are identified as markers for the myoblast differentiation trajectory. Zero-inflated `switchde` fits for these two genes are shown in Figures 1A& B respectively along with the imputed dropout expression (blue crosses). Using the zero-inflated likelihood ratio test these two genes have p-values of 2.371947×10^{-73} and 1.550464×10^{-8}.

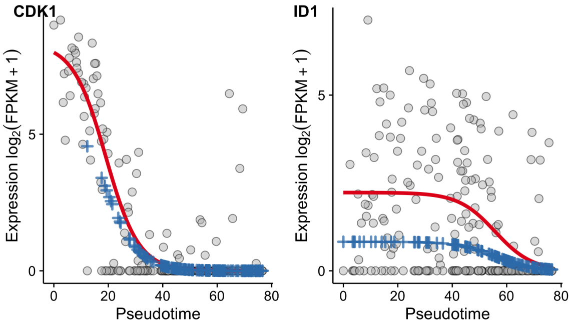

Figure 1: Expression profiles of *CDK1* (A) and *ID1* (B) along with MLE fits for the zero-inflated sigmoidal model (solid red line) with imputed dropout expression (blue crosses).

1.2 Examples of large and small p-values

In Figure 2 we provide example (non-zero-inflated) fits for two genes with drastically different p-values: *NUSAP1* is shown in Figure 2A with a p-value of 5.335782×10^{-69} and *GCLC* with a p-value of 0.9651487. It is clear from the expression plots and MLE sigmoidal fits that the gene with the very low p-value clearly follows the sigmoidal switch-like trend while the gene with the high p-value is well explained by a null (constant expression) model.

1.3 Tracing activation times along pseudotime

One advantage of our model is that one can identify genes up or down regulated at a particular part of the trajectory. To demonstrate this we found the genes with the closest t_0 values to 20%, 32%, 44%, 56%, 68% and 80% of the way through the trajectory, based on all genes significant at 1% FDR.

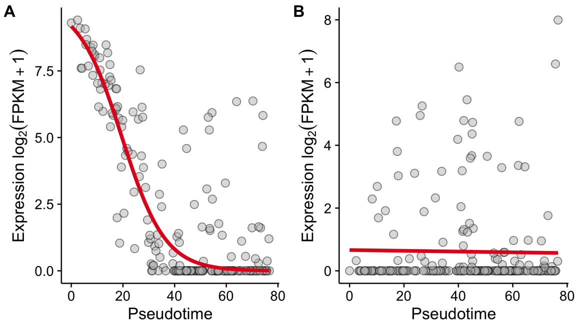

Figure 2: (A) Expression of *NUSAP1* with a p-value of 5.335782×10^{-69} and (B) expression of *GCLC* with a p-value of 0.9651487.

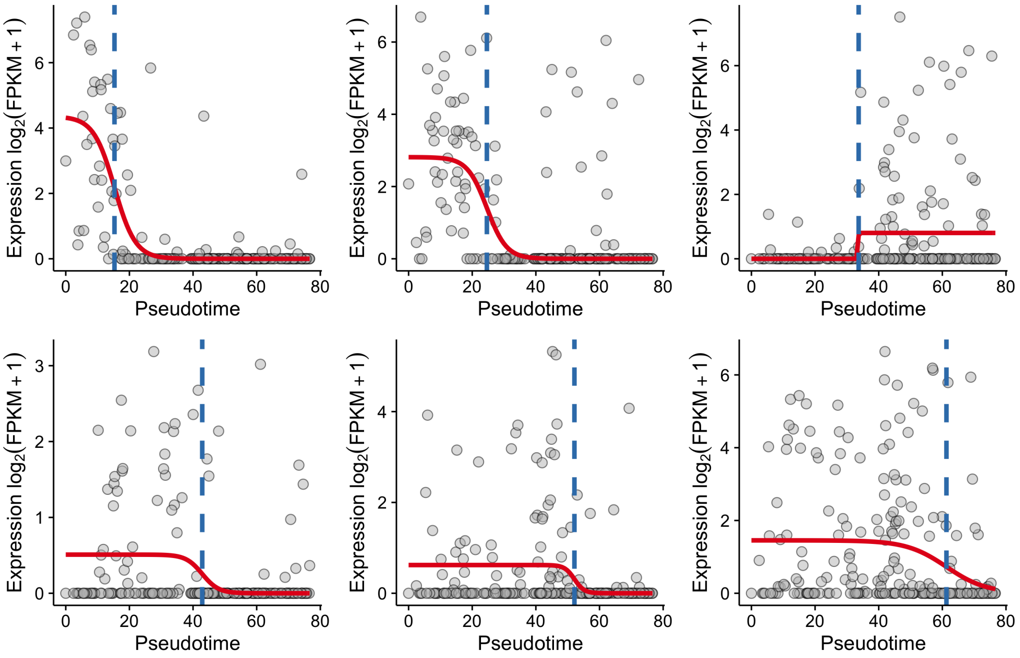

Figure 3: A 'cascade' of gene regulation along pseudotime. Each red curve corresponds to the MLE sigmoidal fit while the vertical blue dashed line corresponds to the MLE of t_0.

The results can be seen in Figure 3, showing a clear 'cascade' of successive gene expression (in)-activations along the trajectory. This also sets the groundwork for identifying temporal gene networks along pseudotime as one can tell whether a given gene is regulated before another.

1.4 Comparison of zero-inflated and standard models

We sought to demonstrate the differences in parameter estimation when considering zero-inflation or otherwise by subsampling 200 genes and fitting both models for each. Figure 4A shows the comparisons of μ_0, demonstrating the zero-inflated estimate is typically higher which agrees with intuition. Figure 4B demonstrates that estimates of k are well calibrated between models. Figure 4C shows the comparison of t_0 estimates which is most dissimilar, with a spike in the non-zero-inflated model corresponding to when t_0 values barely deviate from their initial estimates. Finally, Figure 4D compares the p-value estimates, which shows general concordance with no significant biases for either model.

2 Supplementary methods

2.1 Maximum likelihood model fitting

We begin with a $C \times G$ expression matrix $\mathbf{Y}$ for G genes and C cells with column vector $\mathbf{y}_g, g \in 1, \ldots, G$, that is non-negative and represents gene expression in a form comparable to $\log(\text{TPM} + 1)$. If the sigmoid function

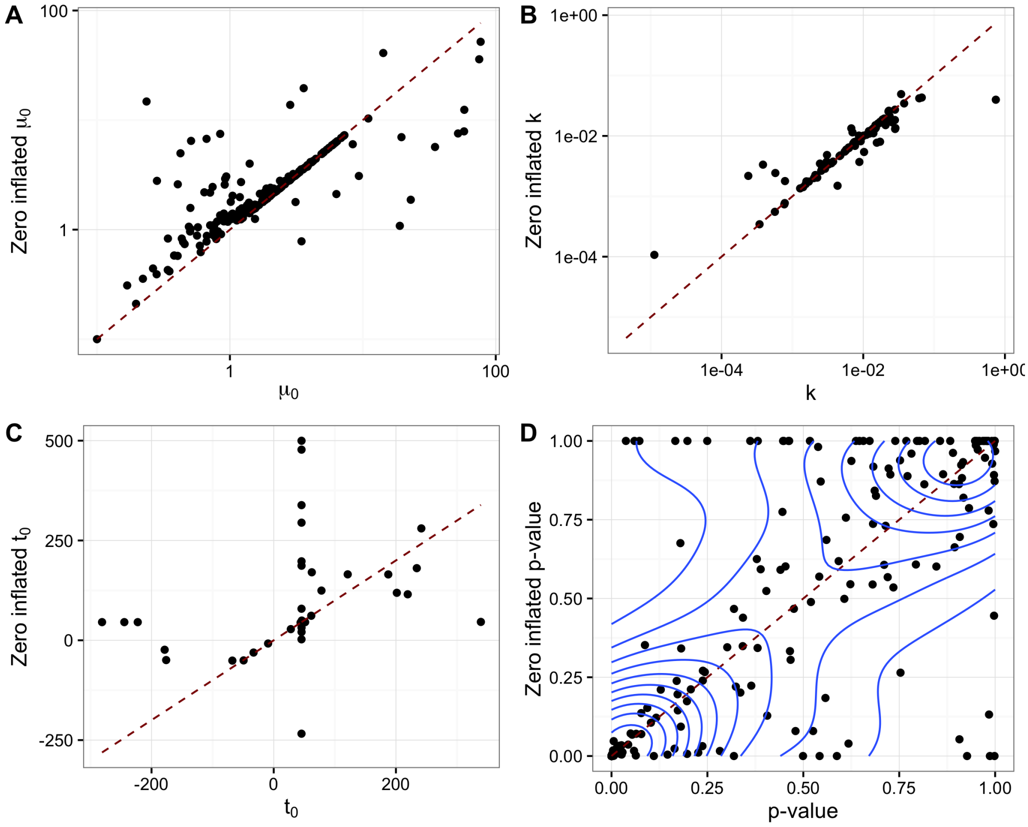

Figure 4: Comparison of MLE parameter estimates for zero-inflated and standard models, for (A) comparison of μ_0, (B) comparison of k, (C) comparison of t_0 and (D) comparison of p-values.

is defined as

$$f(t_c; \mu_g^{(0)}, k_g, t_g^{(0)}) = \frac{2\mu_g^{(0)}}{1 + \exp\left(-k_g(t_c - t_g^{(0)})\right)} \tag{1}$$

then the likelihood of the data given the parameters is

$$L(\mathbf{y}_g, \mathbf{t}; \mu_g^{(0)}, k_g, t_g^{(0)}) = \prod_{c=1}^{C} \mathcal{N}\left(y_{cg} | f(t_c; \mu_g^{(0)}, k_g, t_g^{(0)}), \sigma_g^2\right) \tag{2}$$

We infer maximum likelihood estimates of the parameters using L-BFGS-B optimisation [Byrd *et al.*, 1995] using the `R` function `optim`. This allows fast inference by passing analytical gradients as well as handling constraints on bounded variables. All parameters are defined on $\mathbb{R}$ except for μ_0 and σ^2 which are optimised on $\mathbb{R}^+$. The parameters are initialised as follows: μ_0 is set to $\frac{1}{C}\sum_c y_c$, $t_0 = \text{median}_c\ [t_c]$, $\sigma^2 = \text{Var}_c[y_c]$. We initialise k by using the gradient of the regression of $\mathbf{y}$ off $\mathbf{t}$ to ensure the sign is correct.

We next need to compute the gradients for all parameters. If we consider the function (dropping g subscripts)

$$f(t_c; k, \mu_0, t_0) = \frac{2\mu_0}{1 + \exp\left(-k(t_c - t^{(0)})\right)} \tag{3}$$

(which we may write succinctly as $f(t_c; \Theta)$ where $\Theta = \{\mu_0, k, t^{(0)}\}$) then the partial derivatives are given by

$$\begin{aligned} \frac{\partial f}{\partial \mu_0} &= \frac{2}{1 + \exp\left(-k(t_c - t^{(0)})\right)} \\ \frac{\partial f}{\partial k} &= \frac{f(t_c; \Theta)(t_c - t^{(0)})}{1 + e^{k(t_c - t^{(0)})}} \\ \frac{\partial f}{\partial t^{(0)}} &= \frac{-k f(t_c; \Theta)}{1 + e^{k(t_c - t^{(0)})}} \end{aligned} \tag{4}$$

To find the maximum likelihood estimate of $\Theta \equiv (\mu_0, k, t^{(0)})$ we wish to minimise the negative log-likelihood, given by

$$
\begin{aligned}
\mathcal{L} = -\log L(\mathbf{y}, \mathbf{t}; \Theta) &= -\sum_{c=1}^{C} \log \mathcal{N}\left(y_c | f(t_c; \Theta), \sigma^2\right) \\
&= -\sum_c \left[\log \frac{1}{\sqrt{2\pi\sigma^2}} - \frac{1}{2\sigma^2}\left(y_c - f(t_c; \Theta)\right)^2\right] \\
&= C \log \sqrt{2\pi\sigma^2} + \frac{1}{2\sigma^2} \sum_c \left(y_c - f(t_c; \Theta)\right)^2
\end{aligned} \tag{5}
$$

Then to compute the gradient of $\mathcal{L}$ with respect to a given parameter $\theta \in \Theta$ it follows that

$$
\frac{d\mathcal{L}}{d\theta} = \frac{1}{2\sigma^2} \sum_c \left[-2\left(y_c - f(t_c; \Theta)\right) \frac{\partial f(t_c; \Theta)}{\partial \theta}\right] \tag{6}
$$

Note that this is general to any iid Gaussian measurements with a parametric mean function. Further, since we have

$$
\mathcal{L} \propto \frac{C}{2} \log \sigma^2 + \frac{1}{2\sigma^2} \sum_c \left(y_c - f(t_c; \Theta)\right)^2 \tag{7}
$$

it follows that

$$
\frac{d\mathcal{L}}{d\sigma^2} = \frac{C}{2\sigma^2} - \frac{1}{2\sigma^4} \sum_c \left(y_c - f(t_c; \Theta)\right)^2. \tag{8}
$$

2.2 Expectation-Maximisation for zero inflation

Single-cell RNA-seq data is known to exhibit an over-representation of zeros known as "dropouts". To account for this we propose a model that incorporates dropouts in a similar style to [Pierson and Yau, 2015]. Our model becomes

$$
\begin{aligned}
\mu(t_c, \theta) &= \frac{2\mu_0}{1 + \exp\left(-k(t_c - t^{(0)})\right)} \\
x_c &\sim \mathcal{N}(\mu(t_c, \theta), \sigma^2) \\
h_c | x_c &\sim \text{Bernoulli}(\exp(-\lambda x_c^2)) \\
y_c &= \begin{cases} x_c, & \text{if } h_c = 0 \\ 0, & \text{if } h_c = 1 \end{cases}
\end{aligned} \tag{9}
$$

Where we have replaced $f \to \mu$ to avoid cluttering notation later. We essentially introduce a latent variable x_c for each gene expression measurement but must now perform inference using the Expectation-Maximisation (EM) algorithm due to the intractability of directly maximising the log-likelihood. The secondary latent variable h_c is a binary indicator for whether the expression measurement in cell c is dropout or not. In the following we derive the EM algorithm which follows a similar derivation to [Pierson and Yau, 2015] but with some differences, so it is provided in full below.

In the following let $\Theta = \{\mu_0, k, t^{(0)}, \sigma^2\}$ and consider the complete-data likelihood:

$$
\begin{aligned}
p(\mathbf{y}, \mathbf{x}, \mathbf{h}, \Theta) &= \prod_c p(y_c, x_c, h_c, \Theta), \\
&= \prod_c p(y_c | x_c, h_c) p(h_c | x_c, \Theta) p(x_c | \Theta), \\
p(y_c, x_c, h_c, \Theta) &= \begin{cases} (1 - e^{-\lambda x_c^2}) p(y_c | x_c, h_c = 0) p(x_c | \Theta), & h = 0, \\ e^{-\lambda x_c^2} p(y_c | x_c, h_c = 1) p(x_c | \Theta), & h = 1. \end{cases}
\end{aligned} \tag{10}
$$

We then use the same trick as [Pierson and Yau, 2015]: if $y_c = 0$ then we know necessarily that $h_c = 1$ as $h_c = 0$ with zero probability. Similarly, if $y_c > 0$ then we observe $x_c = y_c$ and know that $h_c = 0$. We therefore split the product up into terms involving $y_c = 0$ and those involving $y_c > 0$, and now consider the log of the complete data likelihood with the shorthand notation $\mu_c \equiv \mu(t_c, \theta)$:

$$\begin{aligned}\mathcal{L}(\mathbf{y},\mathbf{x},\mathbf{h},\Theta) &= \sum_{c:y_c=0}\left[\log\mathcal{N}(x_c|\mu_c,\sigma^2)-\lambda x_c^2\right]+\sum_{c:y_c>0}\left[\log\mathcal{N}(y_c|\mu_c,\sigma^2)+\log(1-e^{-\lambda y_c^2})\right]\\ &= \frac{-C}{2}\log(2\pi\sigma^2)+\sum_{c:y_c=0}\left[\frac{(x_c-\mu_c)^2}{2\sigma^2}-\lambda x_c^2\right]+\sum_{c:y_c>0}\left[\frac{(y_c-\mu_c)^2}{2\sigma^2}+\log(1-e^{-\lambda y_c^2})\right]\\ &= \frac{-C}{2}\log(2\pi\sigma^2)+\sum_{c:y_c=0}\left[-(\frac{1}{2\sigma^2}+\lambda)x_c^2+\frac{\mu_c}{\sigma^2}x_c-\frac{\mu_c^2}{2\sigma^2}\right]+\sum_{c:y_c>0}\left[\frac{(y_c-\mu_c)^2}{2\sigma^2}+\log(1-e^{-\lambda y_c^2})\right]\end{aligned} \tag{11}$$

In order to perform EM we need to calculate the expected value of this log likelihood, conditional on the data $\mathbf{y}$ and a previous estimate $\Theta^{(t)}$:

$$Q(\Theta|\Theta^{(t)})=\mathbb{E}_{\mathbf{x}|\mathbf{y},\Theta^{(t)}}[\mathcal{L}(\mathbf{y},\mathbf{x},\mathbf{h},\Theta)] \tag{12}$$

In order to calculate this it is obvious from equation 11 we must calculate $\mathbb{E}_{\mathbf{x}|\mathbf{y},\Theta^{(t)}}[x_c]$ and $\mathbb{E}_{\mathbf{x}|\mathbf{y},\Theta^{(t)}}[x_c^2]$. Notice we only care about $c:y_c=0$ since for $c:y_c>0$ we know x_c exactly. Note that in all the following all the parameters are assumed fixed at the previous iteration, e.g. $\mu_c\equiv\mu_c^{(t)}$. If we consider a conditional density of the form

$$f(\mathbf{x}|\mathbf{y},\Theta^{(t)})=\prod_c f(x_c|y_c,\Theta^{(t)}) \tag{13}$$

then

$$\begin{aligned}f(x_c|y_c,\Theta^{(t)}) &= \frac{f(y_c|x_c,\Theta^{(t)})f(x_c|\Theta^{(t)})}{\int dx_c f(y_c|x_c,\Theta^{(t)})f(x_c|\Theta^{(t)})}\\ &= \frac{e^{-\lambda x_c^2}\mathcal{N}(x_c|\mu_c,\sigma^2)}{\int dx_c e^{-\lambda x_c^2}\mathcal{N}(x_c|\mu_c,\sigma^2)}\end{aligned} \tag{14}$$

Some algebra later and we arrive at

$$f(x_c|y_c,\Theta^{(t)})=\mathcal{N}\left(x_c|\alpha(t_c,\Theta^{(t)}),\beta(\Theta^{(t)})\right) \tag{15}$$

where

$$\begin{aligned}\alpha(t_c,\Theta^{(t)}) &= \frac{\mu_c}{2\sigma^2\lambda+1}\\ \beta(t_c) &= \frac{\sigma^2}{2\sigma^2\lambda+1}\end{aligned} \tag{16}$$

and so

$$\begin{aligned}\mathbb{E}_{\mathbf{x}|\mathbf{y},\Theta^{(t)}}[x_c] &= \alpha(t_c,\Theta^{(t)})\\ \mathbb{E}_{\mathbf{x}|\mathbf{y},\Theta^{(t)}}[x_c^2] &= \alpha(t_c,\Theta^{(t)})^2+\beta(\Theta^{(t)}).\end{aligned} \tag{17}$$

We need to maximise

$$\begin{aligned}Q(\Theta|\Theta^{(t)}) &= \frac{-C}{2}\log(2\pi\sigma^2)\\ &+\sum_{c:y_c=0}\left[-(\frac{1}{2\sigma^2}+\lambda)\mathbb{E}_{\mathbf{x}|\mathbf{y},\Theta^{(t)}}[x_c^2]+\frac{\mu_c}{\sigma^2}\mathbb{E}_{\mathbf{x}|\mathbf{y},\Theta^{(t)}}[x_c]-\frac{\mu_c^2}{2\sigma^2}\right]\\ &+\sum_{c:y_c>0}\left[\frac{(y_c-\mu_c)^2}{2\sigma^2}+\log(1-e^{-\lambda y_c^2})\right]\end{aligned} \tag{18}$$

with respect to $\theta=\{\mu_0,k,t_0\}$, σ^2 and λ, recalling $\mu_c\equiv\mu_c(\theta,t_c)$. We wish to use gradient-based optimisation and so require the gradients. Note that

$$\begin{aligned}\frac{dQ}{d\theta} &= \sum_c\frac{\partial Q}{\partial\mu_c}\frac{d\mu_c}{d\theta}\\ &= \frac{1}{\sigma^2}\left[\sum_{c:y_c=0}\left(\mathbb{E}_{\mathbf{x}|\mathbf{y},\Theta^{(t)}}[x_c]-\mu_c\right)\frac{d\mu_c}{d\theta}+\sum_{c:y_c>0}(y_c-\mu_c)\frac{d\mu_c}{d\theta}\right]\end{aligned} \tag{19}$$

where the derivatives $\frac{d\mu_c}{d\theta}$ are the same as those given in equation 4. Finally we require the partial derivatives with respect to λ and σ^2 which are given by

$$\frac{dQ}{d\lambda} = -\sum_{c:y_c=0} \mathbb{E}_{\mathbf{x}|\mathbf{y},\Theta^{(t)}}[x_c] + \sum_{c:y_c>0} \frac{y_c^2 e^{-\lambda y_c^2}}{1-e^{-\lambda y_c^2}} \tag{20}$$

and

$$\frac{dQ}{d\sigma^2} = \frac{-C}{2\sigma^2} + \frac{1}{2\sigma^4}\left[\sum_{c:y_c=0} \left(\mathbb{E}_{\mathbf{x}|\mathbf{y},\Theta^{(t)}}[x_c^2] - 2\mu_c \mathbb{E}_{\mathbf{x}|\mathbf{y},\Theta^{(t)}}[x_c] + \mu_c^2\right) + \sum_{c:y_c>0}(y_c-\mu_c)^2\right] \tag{21}$$

Note that σ^2 has an analytical maximum by setting $\frac{dQ}{d\sigma^2}=0$, but since this depends on θ and vice versa we instead numerically optimise all simultaneously.

References

[Byrd *et al.*, 1995] Byrd,R.H., Lu,P., Nocedal,J. and Zhu,C. (1995) A limited memory algorithm for bound constrained optimization. *SIAM Journal on Scientific Computing,* **16** (5), 1190–1208.

[Pierson and Yau, 2015] Pierson,E. and Yau,C. (2015) ZIFA: Dimensionality reduction for zero-inflated single-cell gene expression analysis. *Genome Biology,* **16** (1), 1.

[Trapnell *et al.*, 2014] Trapnell,C., Cacchiarelli,D., Grimsby,J., Pokharel,P., Li,S., Morse,M., Lennon,N.J., Livak,K.J., Mikkelsen,T.S. and Rinn,J.L. (2014) The dynamics and regulators of cell fate decisions are revealed by pseudotemporal ordering of single cells. *Nature Biotechnology,* **32** (4), 381–386.
